# Dr. Charles A. Kingsbury, Philadelphia Dental Club

**Published:** 1892-01

**Authors:** Daniel Neall McQuillen

**Affiliations:** Secretary


### Obituary.

### DR. CHARLES A. KINGSBURY, PHILADELPHIA DENTAL CLUB.

At a meeting of the Philadelphia Dental Club, held November 28, 1891, it was resolved that,—

Whereas, Dr. Charles A. Kingsbury, who had been a member of our Association from its organization in 1872, and who became endeared to us by his high intelligence, the goodness of his heart, and the probity of his character, has, under the Divine providence, been removed from this life, and that we unite with his friends and relatives in mourning his departure, and will keep in remembrance his cheerful Christian influence. Also,—

*Resolved*, That this note be engrossed upon the records of the club, and a copy be sent to his family, and also to the dental journals for publication.

Daniel Neall McQuillen,
*Secretary*.

